# Culturally relevant stressors as moderators of intergenerational transmission of mother-adolescent executive function in Mexican immigrant families

**DOI:** 10.1186/s41235-021-00333-x

**Published:** 2021-11-03

**Authors:** Su Yeong Kim, Jiaxiu Song, Wen Wen, Shanting Chen, Minyu Zhang, Jinjin Yan, Belem G. Lopez, Maria M. Arredondo, Ka I. Ip

**Affiliations:** 1 Department of Human Development and Family Sciences, The University of Texas at Austin, 108 East Dean Keeton St., Stop A2702, Austin, TX 78712 USA; 2 Department of Mexican American and Latina/O Studies, The University of Texas at Austin, Austin, TX USA; 3 Department of Psychology, Yale University, New Haven, CT USA

**Keywords:** Executive functioning, Intergenerational transmission, Economic stress, Foreigner stress, Mexican immigrant family

## Abstract

The intergenerational transmission of executive function may be enhanced or interrupted by culturally salient environmental stressors that shape the practice of executive function in the family. Building upon past research, the current study tests whether culturally relevant stressors such as economic stress and foreigner stress have a direct effect on adolescent executive function, as well as whether they modify the intergenerational transmission of mother–child executive function (i.e., working memory, inhibitory control, and shifting) in low-income Mexican immigrant families. The sample consists of 179 Mexican American adolescents (M_age_ = 17.03 years; SD_age_ = 0.83; 58% females) and their Mexico-born mothers (M_age_ = 43.25 years; SD_age_ = 5.90). Results show that mothers’ perceived economic stress is associated with poor inhibitory control in adolescents. Low levels of mothers’ perceived foreigner stress related to a stronger association between mothers’ and adolescents’ working memory, while high levels of mothers’ perceived foreigner stress related to enhanced intergenerational transmission of poor shifting ability. Study findings demonstrate the prominence of perceived foreigner stress as a contextually relevant factor moderating the intergenerational transmission of mother–child executive function in low-income Mexican immigrant families.

**Supplementary Information:**

The online version contains supplementary material available at 10.1186/s41235-021-00333-x.

## Public Significance Statement

Executive function is represented by a set of cognitive skills critical for children’s academic achievement, health, and well-being. Caregivers play an important role in the development of children’s executive function, not only through shared genes, but also through the environment they provide. Household chaos, for example, has been shown to increase the similarity in executive function between parents and children. However, less is known about how culturally relevant factors for children in immigrant households influence the intergenerational transmission of executive function. Moreover, the bulk of research on executive function focuses on early childhood, with less attention paid to adolescence. Using a sample of Mexico-origin mothers and adolescents from immigrant families, we test two culturally relevant factors, maternal perceived economic stress and foreigner stress, as direct factors in adolescent executive function, and as moderating factors in the intergenerational transmission of executive function. Maternal working memory was significantly associated with adolescent working memory, and high maternal economic stress was associated with worse adolescent inhibitory control. Moreover, low foreigner stress was related to a stronger association between mothers’ and adolescents’ working memory, while high foreigner stress was related to a stronger association between mothers’ and adolescents’ shifting ability. The study highlights how culturally relevant factors, such as maternal perceived foreigner stress, play a significant role in the development of executive function among adolescents. The continued anti-immigrant sentiment and policies in the U.S. are poised to have harmful downstream effects in the development of executive function among adolescents from immigrant families.

## Introduction

Executive function comprises a set of cognitive mechanisms necessary for holding information in one’s mind and manipulating it (i.e., working memory), controlling one’s attention to override external lures (i.e., inhibitory control), and flexibly switching (i.e., shifting) between tasks (Diamond, 2013). Executive function is a set of foundational skills that are necessary for successful academic, behavioral, and socio-emotional development (Diamond, 2013). As caregivers, mothers are influential in the development of children’s executive functioning skills (Kim et al., 2017). Deater-Deckard (2014) further contends that the intergenerational transmission of executive function from parent to child can be altered by contextual features of the home environment (e.g., low socioeconomic status, cultural factors, household chaos). For example, the disorderly environment of chaotic households undermines the practice of executive function in mothers, which is likely to result in poor executive function in adolescents. Moreover, for families with lower socioeconomic status, the overlap of cognitive aptitude in parent–child dyads is more sensitive to environmental factors (Tucker-Drob et al., 2013). We extend this research on the overlap of cognitive aptitude in low-income families, as moderated by environmental factors, to the intergenerational transmission of executive function in mother-adolescent dyads among low-income Mexican immigrant families.

As a group, low-income Mexican children of immigrants are more likely to have less-educated parents, live in poverty, and lag behind their peers on school outcomes (Tienda & Haskins, 2011). Mexican immigrant families also experience foreigner stress (Kim, Hou, et al., 2018a; Zou & Cheryan, 2017), which is the belief that others perceive them as foreigners due to factors such as their ethnic minority status, appearance, or having an accent while speaking English (Armenta et al., 2013). Collectively, then, Mexican immigrants in the U.S. may be a cultural group particularly vulnerable to both economic and foreigner stress (Armenta et al., 2013; Kim, Schwartz, et al., 2018b). There is evidence to suggest that culturally relevant stressors can undermine cognitive abilities, such as executive function (Ozier et al., 2019).

We propose to test how culturally relevant stressors (i.e., economic and foreigner stress) in the environment influence the intergenerational transmission of executive function between mothers and adolescents in Mexican immigrant families. Our focus is due to the paucity of research on executive function in adolescence specifically, and limited research on this topic as it pertains to adolescents from low-income and ethnic minority backgrounds. Adolescence is a developmental period that is susceptible to the effects of stress (Tottenham & Galván, 2016), and executive function continues to develop throughout adolescence and into young adulthood (e.g., Blakemore & Mills, 2014; Theodoraki et al., 2019). To address this gap in knowledge, our study examines the development of executive function components (i.e., inhibitory control, working memory and shifting) through mother-adolescent intergenerational transmission, and examines how *perceived economic stress* and *perceived foreigner stress* may influence adolescent executive function, and may also moderate (e.g., exacerbate or enhance) the intergenerational transmission of executive function.

### Intergenerational transmission of executive function

Intergenerational transmission of executive function refers to the familial transmission of executive function from parent to child, which is manifested in the correlation between parent and child executive function (Brieant et al., 2017; Jester et al., 2009). Such intergenerational transmission has been documented as a potential source of individual variability in children’s executive function (Bridgett et al., 2015; Deater-Deckard, 2014), and the effect of intergenerational transmission of executive function persists into adolescence (Brieant et al., 2017; Jester et al., 2009). Indeed, Jester et al. (2009) found that mothers’ executive function was positively associated with their adolescent children’s executive function, even after accounting for parental and child IQ. Parents influence their children’s executive function not only by the intergenerational transmission of genes, but also through the home environment in which their children are reared (Lewis & Carpendale, 2009; Sosic-Vasic et al., 2017). For example, parents can set household rules (e.g., discourage adolescents from using a mobile phone during family meal times) as a way for adolescents to practice inhibitory skills, and adolescents practice their memory and attention skills while communicating with parents. Moreover, given that mothers are often the primary caregivers, adolescents may spend more time with their mothers. The frequency of mother-adolescent interactions within the shared family environment may enable the intergenerational transmission of executive function from mother to adolescent. To understand individual variability in adolescent executive function, then, it is important to consider the factors in the rearing environment which may affect adolescents’ executive function and the strength of intergenerational transmission of executive function from mother to child (Deater-Deckard, 2014).

In the current study, we focused on three specific executive functions: inhibitory control, working memory, and shifting ability. Lower inhibitory control is associated with higher likelihood of engaging in risky behaviors (e.g., substance use;Heitzeg et al., 2015). Meanwhile, as adolescents are surrounded with distractions like media and online games, enhanced shifting ability may help them shift attention from external lures to academic tasks, and better working memory may enable them to store and manipulate information needed for academic work. In fact, previous studies have found that the ability to shift attention and possessing strong working memory are key executive function skills for academic success among adolescents (Li et al., 2020; Rigoli et al., 2012). Hence, understanding factors that are associated with heterogeneity in executive function is important, given that adolescence is a period with increased incidence of risk-taking behaviors (Tymula et al., 2012), and academic outcomes during this period predict long-term adult outcomes (Lê-Scherban et al., 2014).

### Environmental stressors and different types of executive function

Environmental stressors create differential opportunities for specific types of executive function to be more or less adaptive. For example, in highly stressful environments, inhibitory skills are undermined while shifting abilities are enhanced (Mittal et al., 2015), suggesting that some types of executive function (i.e., shifting) are adaptive when faced with high levels of environmental stressors. Specifically, according to the adaptation-based approach to resilience (Ellis et al., 2017), living in a chaotic household, a source of environmental stress, amplifies shifting skills due to the need to detect threats and short-lived opportunities in an unpredictable environment. At the same time, a chaotic household may diminish inhibitory control due to a distracting environment diverting an individual's attention, making it more difficult to suppress temptations for external stimuli (Diamond, 2013). Additionally, exposure to environmental stress disrupts working memory by impeding the ability to hold information in the short term in one’s mind (Luethi et al., 2009). Overall, specific executive function may be strengthened or undermined by an environmental stressor in terms of whether the executive function is adaptive in the context of the stressor.

### Environmental stressors and intergenerational transmission of executive function

Based on the premise of the transactional model of cognitive development (Tucker-Drob et al., 2013), for children from families with enriched environments (as indexed by high socio-economic status), heritability accounts for most of the variance in intergenerational transmission of cognitive aptitude, with environmental factors showing a minimal influence (Tucker-Drob et al., 2013). In contrast, in lower socioeconomic status (SES) families, the intergenerational transmission of cognitive ability is demonstrated through a stronger influence of the environment (i.e., socioeconomic disadvantages) repressing the genetic effect (Deater-Deckard et al., 2012). This suggests that studies on low-SES families need to take into consideration the potential role of environmental stressors in explicating the intergenerational transmission of parent–child executive function abilities. In fact, Brieant et al. (2017) demonstrated that household chaos, which is characteristic of low-income families, may increase intergenerational transmission of pre-existing executive function ability from mother to child. Based on Brieant et al.’s (2017) study, then, in low- income families, children’s executive function may resemble that of parents because environmental stressors are salient in this context, shaping how family members practice their executive function. However, the intergenerational transmission of executive function may not be uniform across different components of executive function.

One important factor yet to explore is whether *specific* executive function components (i.e., inhibitory control, working memory and shifting) may be differentially transmitted across generations, and/or influenced by different environmental stressors. We expect the intergenerational transmission of each type of executive function to be distinct, depending on whether the specific executive function that is practiced is adaptive in a given environment. For example, when mothers encounter environmental stressors (e.g., foreigner stress or economic stress), the intergenerational transmission of working memory and inhibitory control may be undermined, given the obstacles to practicing working memory and inhibitory control abilities in the high-stress context. On the other hand, as enhanced shifting ability is adaptive in high-stress contexts, as it aids in adjusting to changing and uncertain environment (Mittal et al., 2015), the intergenerational transmission of shifting may be enhanced when mothers face environmental stressors.

We explore our research questions using a sample of adolescents, given the emergence of distinct executive function components becoming more pronounced during adolescence than they were earlier in childhood (Bardikoff & Sabbagh, 2017). This is likely due to lengthy maturation periods in brain structures that aid executive function, such as the prefrontal cortex, which matures in adolescence and early adulthood (Choudhury et al., 2006; Lee et al., 2013). We focus on Mexican immigrant families because the only study on how familial stress may influence executive function transmission was based on mostly European American families and did not examine how each component of executive function transmits intergenerationally from mother to child (Brieant et al., 2017). Therefore, it remains unclear how stressors more relevant to culturally distinct groups, such as Mexican immigrant families, operate in mother-adolescent intergenerational transmission of executive function.

### Economic stress as a culturally relevant stressor in Mexican immigrant families

Mexican immigrant families in the U.S. often experience high rates of poverty (Lopez & Velasco, 2011), and economic stress may be a particularly relevant environmental factor for this group. In fact, children from disadvantaged socioeconomic backgrounds often perform worse on executive function measures (Raver et al., 2013; Theodoraki et al., 2019), and a meta-analysis revealed a small to moderate effect size between socioeconomic status and child executive function (Lawson et al., 2018).

Based on the family stress model (Conger & Donnellan, 2007), economic stress—from being unable to pay necessary expenses, having inadequate material means, or experiencing financial strain—can indirectly result in negative developmental outcomes for children due to effects on parental behavior and parenting strategies. Indeed, in low-income Mexican immigrant families, parents’ subjective experience of economic hardship is a proximal predictor of family process and child developmental outcomes (White et al., 2009). Given this premise, we focus on mothers’ perceived economic stress as a culturally relevant factor that may be associated with, and/or moderate, the intergenerational transmission of executive function, even after accounting for mothers’ socioeconomic status (as indicated by mothers’ educational attainment).

### Foreigner stress as a culturally relevant stressor in Mexican immigrant families

In addition to economic stress, immigrant and ethnic minority families often experience stressors related to their daily experiences as minorities. Although Latinos may identify themselves as Americans, much like their European American counterparts, a growing body of research has found that they are often viewed as less American due to their physical appearance and ethnic minority background, or because they speak English with an accent (Armenta et al., 2013). According to the racial position model (Zou & Cheryan, 2017), foreigner stress—a cultural stereotype in which ethnic minorities are seen as foreigners (Armenta et al., 2013)—is a more salient and distinct stressor for Latinos compared to other ethnic minority groups (e.g., African American). Mothers with more perceived foreigner stress may feel alienated in their environment, which may lead to a decrease in motivation and/or lessen their ability to recruit cognitive resources (e.g., working memory) when interacting with their children. No study thus far has examined the potential moderating role of mothers’ foreigner stress on adolescents’ cognitive development (e.g., intergenerational transmission of executive function). However, studies have shown that discriminatory experiences represent a salient stressor that may influence how one performs on executive function tasks (Ozier et al., 2019). If so, foreigner stress as a form of discrimination may need to be considered in testing the intergenerational transmission of parent–child executive function.

Our goal is to examine whether mothers’ foreigner stress is a moderator operating in the intergenerational transmission of Mexican-origin mothers’ executive function to their adolescents. We hypothesize that mothers’ perceived foreigner stress, as a culturally relevant stressor, will moderate the mother-adolescent transmission of executive function in Mexican immigrant families. The potential moderating effect of maternal foreigner stress is particularly important to explore, given that economic stress (mostly indexed as lower SES) is a well-studied ecological stressor related to cognitive development (Raver et al., 2013), but less is known about other culturally-relevant stressors beyond the impact of lower SES, and how they operate in immigrant and ethnic minority families.

## The current study

**Fig. 1:**
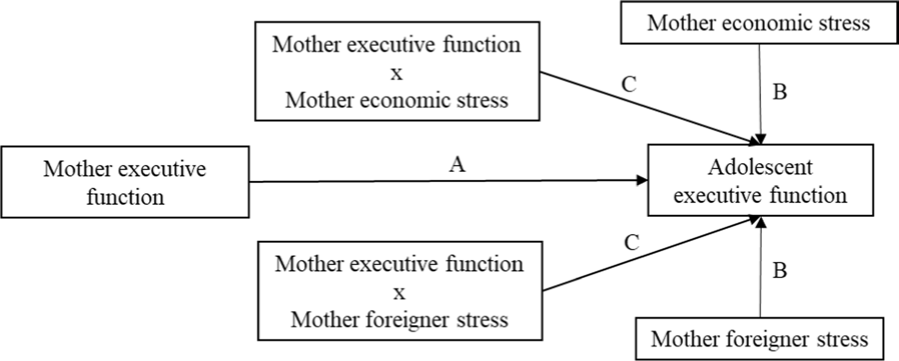
Conceptual model of the moderated intergenerational transmission of executive function. A path indicates the intergenerational transmission of executive function. B paths indicate the main effects of maternal stressors. C paths indicate the moderating effect of maternal stressors. Each executive function component (i.e., working memory, inhibitory control, and shifting) was tested in a separate model

The current study examined mother-adolescent intergenerational transmission of executive function, and potential culturally relevant stressors that may moderate these processes among low-income immigrant Mexican families—a high-risk group that has not been the focus of study in the executive function literature. The first goal is to examine the strength of intergenerational transmission of different executive function components in mother-adolescent dyads from Mexican immigrant families (Path A in Fig. 1). Our second goal is to examine whether mothers’ perceived economic stress and foreigner stress have a direct effect on adolescents’ executive function components (Path B in Fig. 1). Our third goal is to examine whether mothers’ perceived environmental stressors, especially economic stress and foreigner stress, moderate the intergenerational transmission of executive function (Path C in Fig. 1). Specifically, we examine whether mother-adolescent executive function transmission may be strengthened in environments in which mothers’ perceived economic stress and foreigner stress are at low levels, reflecting the idea that low stress is more conducive to the intergenerational transmission of cognitive aptitude from parents to children (Tucker-Drob et al., 2013). No specific hypothesis was made about whether mothers’ perceived economic stress and foreigner stress would differentially impact the intergenerational transmission of different executive function components, due to insufficient evidence to make strong predictions.

## Methods

### Participants

A total of 179 Mexican American bilingual adolescents (M_age_ = 17.03 years; SD_age_ = 0.83; age range = 16–19; 75 males, 104 females) and their Mexico-born mothers (M_age_ = 43.25 years; SD_age_ = 5.90; age range = 35–66) participated in the research. Data collection and recruitment took place around a metropolitan city in central Texas between 2017 and 2018. Approximately 73.7% of the adolescents (*N* = 132) were U.S.-born, while the rest were Mexico-born. The median and mode of mothers’ education level was middle school or junior high school. The median and mean household family income for participants was in the range of $30,001 to $40,000 (see Table 1).

**Table 1:** Correlation and descriptive statistics of study variables

	1	2	3	4	5	6	7	8	9	10	11	12	13	14	15
1. Mother DS	1														
2. Adolescent DS	.20^*^	1													
3. Mother RT of Simon	.02	.01	1												
4. Adolescent RT of Simon	− .10	− .03	− .05	1											
5. Mother RT in SC	.02	− .07	− .07	− .04	1										
6. Adolescent RT in SC	.01	− .10	.11	− .03	.07	1									
7. Mother foreigner stress	.05	− .04	− .05	− .10	.03	− .11	1								
8. Mother Economic stress	.09	.15	− .06	.20^**^	.07	.01	.04	1							
9. Annual family income	.11	.14	− .01	.07	− .05	− .04	− .08	.21**	1						
10. Mother highest educ. level	.26^**^	.10	− .09	− .06	.06	− .18^*^	.09	.31***	.26**	1					
11. Adolescent age	− .12	− .20^*^	− .07	.11	.03	.07	− .20^**^	− .06	− .02	− .02	1				
12. Adolescent gender^a^	.06	.10	.09	− .01	− .01	.03	.09	− .02	− .05	.01	− .06	1			
13. Adolescent nativity^b^	− .06	− .01	− .09	.04	.08	− .06	− .02	− .09	.01	.04	.15	− .01	1		
14. Adolescent Spanish prof.^c^	− .04	.139	.01	.02	.07	− .05	.20^**^	− .09	.07	.06	.05	.17^*^	.32^***^	1	
15. Adolescent English prof.^c^	.13	.056	.06	− .10	− .05	.05	.12	.09	.05	.08	.03	.09	.10	.20^**^	1
*N* (% missing)	133	168	179	179	177	177	178	179	165	179	179	179	179	179	179
	(26%)	(6%)	(0%)	(0%)	(1%)	(1%)	(1%)	(0%)	(8%)	(0%)	(0%)	(0%)	(0%)	(0%)	(0%)
*Mean*	4.58	5.13	81.98	37.89	52.44	119.49	3.31	1.82	3.54	5.51	17.03	58% female	26% Mexican	3.60	4.32
*SD*	1.11	1.03	52.29	42.53	207.35	176.72	0.85	0.20	2.16	2.40	0.83	42% male	74% US	0.70	0.56
*Skewness*	0.59	0.35	0.16	0.77	0.54	0.14	− 0.48	− 1.23	1.38	0.25	0.18	–	–	− 0.39	− 0.47
*Kurtosis*	0.11	0.61	− 0.04	3.51	3.22	1.65	− 0.06	1.12	2.42	− 0.70	− 0.98	–	–	− 0.16	0.02

*DS*, digit span; *RT*, reaction time; *Simon*, Simon effect; *SC*, switch cost; *Educ.*, education; *Prof.*, proficiency. *p < .05, **p < .01, ***p < .001

^a^Adolescent gender is coded as female = 1 and male = 0

^b^Adolescent nativity is coded as Mexican = 1 and US = 0

^c^Spanish and English proficiency were measured using a 5-point scale, ranging from 1 = *not well* to 5 = *extremely well*

#### Materials backward digit span

This task measures individuals’ working memory (Wechsler, 1997), and materials from the Wechsler scale (Macnamara & Conway, 2016) were modified for computer-based presentation. Participants were presented with sets of digit lists (3–9 digits in length, 6 trials per digit length) on the computer screen and were instructed to remember the previously presented digits, then type the digits in the reverse (i.e., backward) order of presentation. For example, if a participant were presented with the digits “829”, then the participant would need to type in “928”. Trials began with a fixation cross (+) presented in the middle of the computer screen (*x* = 6 in., *y* = 3.38 in.), for 3000 ms (ms). Digit span trials were presented on the screen for 1000 ms times the number of digits in the list (e.g., 3000 ms presentation for a 3-digit list, 4000 ms for a 4-digit list, etc.).

Digit span was calculated by taking the score of the last digit list item for which the participant answered *four* out of the six trials correctly. If the participant failed to meet this criterion at a given length, the score from the previous digit span length was used. Participants who did not complete the three-digit practice trials successfully did not receive a score for digit span (25% for mothers, 6% for adolescents). Although some research suggests an accuracy cutoff of answering *five* out of the six trials correctly (Della Sala et al., 2010), using this cutoff resulted in a large number of missing cases in the current low-income immigrant sample. We therefore decided to use the *four-out-of-six* cutoff criterion to retain at least 70% of the data. Higher scores in the backward digit span task indicate better working memory.

#### Simon Task

The Simon Task measures inhibitory control via stimulus–response reactions (Bialystok et al., 2004; Simon & Rudell, 1967). Each participant completed 32 trials (16 congruent trials, 16 incongruent trials) in a randomized order. Each trial began with an 800-ms fixation cross (+) at the screen center (*x* = 6 in., *y* = 3.38 in.), followed by a 250-ms blank interval. Then, a stimulus appeared on the left (*x* = 3 in., *y* = 3.38 in.) or right (*x* = 9 in., *y* = 3.38 in.) side of the screen for 1,000 ms or until a response was made, followed by another 250-ms blank interval. Participants were instructed to press the keys on the keyboard that corresponded to the colors presented on the screen as quickly and as accurately as possible, using their index fingers. For congruent trials, the red/blue squares were presented to the same side as the corresponding keyboard key (e.g., the red square was presented on the left and the blue square was presented on the right). For incongruent trials, the stimuli were presented on the opposite side of the corresponding keyboard key.

#### Color-Shape Task

The Color-Shape Task measures cognitive shifting via stimulus–response reactions (Mittal et al., 2015; Miyake et al., 2004). A total of 48 trials (24 stay trials, 24 switch trials) were randomized and presented to participants in the same order. At the beginning of each trial, the target word “COLOR” or “SHAPE” appeared first on the screen for 200 ms in black at the top of the white screen (*h* = 0.357-in., font style Courier in size 14). Next, an image of a triangle (*h* = 2.93 in., *w* = 3 in.) or circle (*d* = 3 in.) appeared, with the same target word on the top of the screen. The stimulus word and image remained on the screen until the participant selected a response key. A 600-ms blank slide was presented between trials. Using their index fingers, participants categorized the images according to the given target word, as quickly and as accurately as possible, by pressing keys on the keyboard. For a stay trial, the given categorization word was consistent with the previous trial (i.e., “SHAPE” to “SHAPE”, “COLOR” to “COLOR”). For a switch trial, the given categorization word was different from the previous trial (i.e., “SHAPE” to “COLOR”, “COLOR” to “SHAPE”).

For both the Simon and Color-Shape tasks, response accuracy and reaction time in milliseconds were recorded. For both adolescents and mothers, the accuracy of the congruent (adolescent*: *Mean = 97.5%, mother: Mean = 98.7%) trials in the Simon Task were almost at ceiling, as was the accuracy of the stay (adolescent: Mean = 94.6%, mother: Mean = 85.6%) trials in the Color-Shape Task. Therefore, we elected to use only the reaction time as the indicator of performance for both tasks (Weintraub et al., 2013). The Simon effect was calculated for each participant by subtracting their mean reaction time for congruent trials from their mean reaction time for incongruent trials, with shorter time indicating better inhibitory control. The switch cost was calculated for each participant by subtracting their mean reaction time for stay trials from their mean reaction time for switch trials, with shorter time denoting better shifting.

### Measures

#### Mother foreigner stress

Foreigner stress was assessed using a four-item scale validated among Mexican-origin adolescents (e.g., “Because of how I speak, people sometimes assume I am not a U.S. American”, Kim, Hou, et al., 2018a). Mothers rated each item on a 5-point scale, ranging from *1* = *strongly disagree* to *5* = *strongly agree*. Scores were averaged across the four items so that higher scores indicate greater foreigner stress (Cronbach’s alpha = 0.80).

#### Economic stress

Economic stress was measured with nine dichotomized items adapted from Conger et al. (2002). Mothers reported whether or not the family experienced any of the specified situations because of financial needs (e.g., “fell far behind in paying bills” and “sold some possessions because you needed the money even though you really wanted to keep them”). Answers (*1* = *No*, *2* = *Yes*) were averaged across the nine items so that higher mean scores indicate greater economic stress (Cronbach’s alpha = 0.68).

#### Covariates

Studies have found evidence that age, gender, immigrant status, and language proficiency have an influence on individuals’ executive function. For example, executive control undergoes a maturation process during the course of adolescence, and demonstrates different kinds of gender differences depending on the specific task (e.g., Malagoli & Usai, 2018). Adolescents of later immigrant generations have demonstrated better shifting ability (e.g., Williams et al., 2019), and bilingualism can undermine as well as benefit an individual’s executive control (Bialystok, 2011; Hilchey & Klein, 2011). Therefore, we included adolescents’ self-reported age, gender, nativity (i.e., foreign-born or U.S.-born), and Spanish and English proficiency as covariates in our study. Specifically, adolescents’ proficiency in reading, writing, speaking, and understanding Spanish and English was assessed using a five-point scale validated for use with Mexican-origin adolescents (Kim et al., 2020), ranging from *1* = *not well* to *5* = *extremely well* (Cronbach’s alphas = 0.81 for Spanish and 0.82 for English). Past studies have found that self-report measures of language proficiency are correlated with objective measures of language proficiency (Dunn & Fox Tree, 2009). We also controlled for mothers’ highest education level, which was found to be positively correlated with child cognitive functioning (Bernier et al., 2010). Mothers’ highest education level was assessed on a 11-point scale ranging from *1* = *no formal schooling* to *11* = *finished graduate degree*.

### Procedure

Participants (both adolescents and mothers) were tested individually during a home visit, using laptops brought by research assistants. All executive function tasks were presented on a laptop (Dell Latitude 3480 14 in.) using the software package E-Prime 2.0 (Schneider et al., 2002). Participants first completed the Backward Digit Span, followed by the Simon Task and Color-Shape Task. For the Simon Task and Color-Shape Task, participants completed four practice trials prior to the experimental trials. After completing the executive function tasks, participants completed online questionnaires about their language background and experiences. All materials were prepared in both English and Spanish. The questionnaires were originally designed in English. A team of bilingual and bicultural research assistants first translated the materials to Spanish and then back-translated them to English. Participants chose which language they preferred to use for the interview and cognitive tasks. Families were compensated $60 for participating in the study.

### Analytical strategy

Missing data rates ranged from 0 to 1% for all variables collected via survey. Indicators of executive function had missing rates ranging from 0 to 25% (see Table 1 for details). Little’s test of missing completely at random (Little, 1998) was conducted in SPSS, demonstrating that any missing data was completely at random (*χ*^2^(62) = 61.86, *p* = 0.48). The following path analyses were conducted in Mplus 7.3 using maximum likelihood estimation with robust standard errors (Muthén & Muthén, 1998–2012) to test the study’s questions. For each participant in the Simon task or the color-shape task, trials with reaction times outside three standard deviations from each person’s mean for each condition (i.e., congruent and incongruent conditions for the Simon task; switch and stay conditions for the color-shape task) were excluded from analysis. As shown in the conceptual model (Fig. 1), a set of baseline models linking mothers’ executive function to adolescents’ executive function were tested for each executive function separately (i.e., working memory, inhibitory control/Simon effect, and shifting/switch cost). We then tested a set of main effect models where the main effects of mothers’ foreigner stress and mothers’ economic stress to adolescents’ executive function were added to the baseline model. Building upon the main effect models, two moderation paths were added to examine whether mothers’ perceived foreigner stress and economic stress moderated the intergenerational transmission of executive function. Missing data were handled using Full Information Maximum Likelihood (FIML). Covariates included mothers’ highest education level, adolescents’ age, gender, nativity, Spanish proficiency, and English proficiency. Effects of covariates on all study variables were controlled in the model. Table 1 presents descriptive information on the study’s variables and demographics. Additional file 1: Figures S1 to S3 are violin plots showing the distribution of adolescents’ and mothers’ performances on each executive function task.

## Results

### Path model results

The baseline models with covariates were saturated models. As shown in Table 2, only the working memory model showed that mothers’ digit span was positively associated with adolescents’ digit span with a significant r-square in explaining the variability in adolescent digit span (*b* = 0.18, SE = 0.08, *p* = 0.03). All main effect models (i.e., when both economic and foreigner stress were added to the baseline model) had adequate model fit (Table 2). Specifically, the working memory model revealed a significant positive main effect of mothers’ digit span on adolescents’ digit span (*b* = 0.18, SE = 0.08, *p* = 0.03), while no direct effects of economic stress or foreigner stress on adolescents’ digit span were found. For the switch cost model, no significant main effect of any predictor on adolescents’ switch cost reaction time was found (Table 2). For the inhibitory control model, there was a positive main effect of mothers’ economic stress on adolescents’ Simon effect (*b* = 38.71, SE = 18.00, *p* = 0.03), indicating that higher economic stress was associated with worse inhibitory control.

**Table 2:** Unstandardized coefficients of baseline models, main effect models, and moderation models

	Model 1: Baseline model			Model 2: Main effect model			Model 3: Moderation model		
	*b*	SE	*p*	*b*	SE	*p*	*b*	SE	*p*
*Working memory model: adolescent digit span ON*									
Mother digit span	**0.18**	**0.08**	**.03**	**0.18**	**0.08**	**.03**	**0.18**	**0.08**	**.03**
Mother foreigner stress	–	–	–	− 0.159	0.10	.10	− 0.16	0.09	.09
Mother economic stress	–	–	–	0.70	0.41	.09	− 0.51	0.39	.19
Mother digit span × FS	–	–	–	–	–	–	− **0.27**	**0.11**	**.01**
Mother digit span × ES	–	–	–	–	–	–	0.65	0.46	.15
*R-Square*	**0.11**	**0.05**	**.00**	0.06	0.03	.06	**0.20**	**0.06**	**.001**
Chi-square test	–			*χ*^2^(3) = 0.24, *p* = .97			*χ*^2^(9) = 6.72, *p* = .67		
CFI	–			1.00			1.00		
RMSEA [90% CI]	-			.00 [90% CI: 0.00, 0.00]			.00 [90% CI: 0.00, 0.07]		
*Shifting model: adolescent reaction time of switch cost ON*									
Mother RT in switch cost	0.08	0.06	.16	0.08	0.06	.17	0.05	0.06	.44
Mother foreigner stress	–	–	–	− 18.58	15.56	.23	− 21.41	15.64	.17
Mother economic stress	–	–	–	− 27.44	74.20	.71	− 30.96	71.90	.67
Mother RT in switch cost × FS	–	–	–	–	–	–	**12.97**	**6.44**	**.04**
Mother RT in switch cost × ES	–	–	–	–	–	–	32.85	28.07	.24
*R-Square*	0.06	0.03	.09	0.06	0.03	.06	**0.08**	**0.04**	**.03**
Chi-square test	–			*χ*^2^(3) = 1.07, *p* = .78			*χ*^2^(9) = 10.28, *p* = .33		
CFI	–			1.000			0.96		
RMSEA [90% CI]	–			.00 [90% CI: 0.00, 0.08]			.03 [90% CI: 0.00, 0.09]		
*Inhibitory control model: adolescent reaction time of Simon effect ON*									
Mother RT in Simon effect	− 0.03	0.06	.65	− 0.04	0.06	.51	− 0.04	0.06	.56
Mother foreigner stress	–	–	–	− 4.12	3.33	.22	− 4.48	3.40	.19
Mother economic stress	–	–	–	**38.71**	**18.00**	**.03**	**40.54**	**18.02**	**.02**
Mother RT in Simon effect × FS	–	–	–	–	–	–	0.10	0.07	.18
Mother RT in Simon effect × ES	–	–	–	–	–	–	0.12	0.33	.73
*R-Square*	0.03	0.03	.25	0.07	0.04	.10	0.08	0.05	.09
Chi-square test	–			*χ*^2^(3) = 1.57, *p* = .67			*χ*^2^(9) = 4.31, *p* = .89		
CFI	–			1.00			1.00		
RMSEA [90% CI]	–			.00 [90% CI: 0.00, 0.10]			.00 [90% CI: 0.00, 0.04]		

All models controlled for six covariates, including mothers’ highest education level, adolescents’ age, gender, nativity, Spanish proficiency, and English proficiency. All significant results are bolded. *FS*, foreigner stress; *ES*, Economic stress

The moderation models fit the data well across the three executive function components (Table 2). In the working memory model, the positive main effect of mothers’ digit span (*b* = 0.18, SE = 0.08, *p* = 0.03) remained significant after adding the two moderating paths. There was also a significant interaction effect of mothers’ digit span and mothers’ foreigner stress (*b* = − 0.27, SE = 0.11, *p* = 0.01) on adolescents’ digit span. While no main effect of adolescents’ switch cost was observed (Table 2), the model for shifting ability revealed a significant interaction term between switch cost reaction time (RT) and mothers’ foreigner stress (*b* = 12.97, SE = 6.44, *p* = 0.04) to adolescents’ switch cost RT. In addition, similar to the main effect model (Table 2), the moderation model for inhibitory control revealed a significant direct positive effect of mothers’ economic stress (*b* = 40.54, SE = 19.01, *p* = 0.02) on adolescents’ Simon effect. Neither foreigner stress nor economic stress showed a significant moderating effect in the inhibitory control model. In addition, it is also worth noting that maternal education level, as an indicator of socio-economic status, did not have a significant positive effect on adolescents’ executive function across the three models (*b* = − 0.12 to 0.04, SE = 0.04 to 1.32, *p* = 0.06 to 0.98).

### Simple slope analyses

**Fig. 2:**
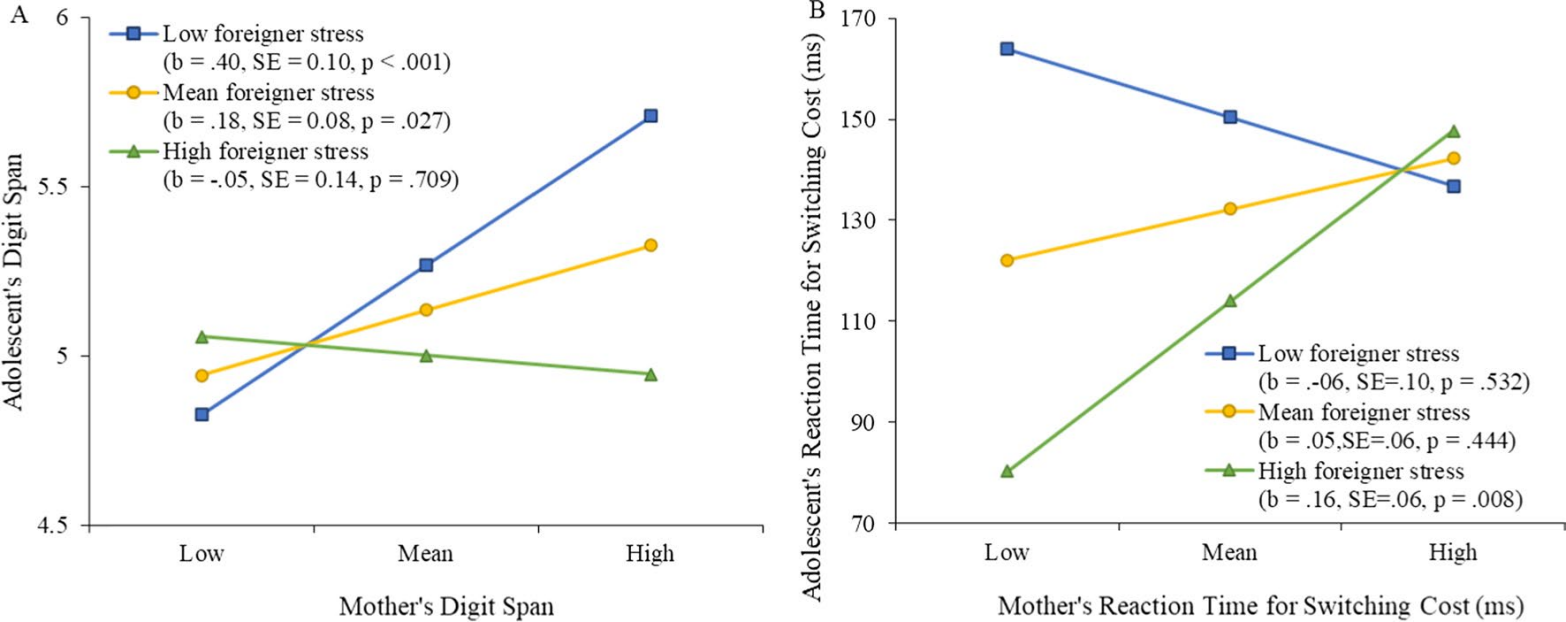
**A** Mothers’ working memory relating to adolescents’ working memory (as indexed by backward digit span, where a larger digit span represents better working memory) at low (− 1 *SD*), mean, and high (+ 1 *SD*) levels of maternal foreigner stress. **B** Mothers’ shifting ability (as indexed by the reaction time of switching cost in the Color-Shape task in milliseconds, where a shorter reaction time for shifting cost represents better shifting ability) relating to adolescents’ shifting ability at varying levels of maternal foreigner stress. The numbers in parentheses indicate unstandardized simple slopes and their *p*-value

Simple slope analyses were performed to probe the two significant interactions at the mean level, one standard deviation below the mean (low), and one standard deviation above the mean (high) for each moderator. For the interaction of mothers’ digit span and mothers’ foreigner stress with adolescents’ digit span, the analysis (Fig. 2A) showed that a larger maternal digit span was significantly related to larger adolescent digit span *only* when mothers reported low (− 1 SD) levels (*b* = 0.40, SE = 0.10, *p* < 0.001) or mean levels of foreigner stress (*b* = 0.18, SE = 0.08, *p* = 0.03) but not high (+ 1 SD) levels of foreigner stress. Probing the switch cost RT × mothers’ foreigner stress interaction (Fig. 2B) showed that mothers’ RT in switch cost was positively associated with adolescents’ RT in switch cost *only* when mothers reported high levels of foreigner stress (*b* = 0.16, SE = 0.06,* p* = 0.008) but not mean or low levels.

## Discussion

The link from parent executive function to child executive function has been demonstrated mainly in early childhood (Deater-Deckard, 2014). More recently, studies have found that the intergenerational transmission of executive function could persist into early adolescence (i.e., ages 12–14; Brieant et al., 2017; Jester et al., 2009). Our study extends previous findings by observing intergenerational transmission of executive function components (i.e., shifting, working memory) in late adolescence (i.e., ages 16–19), suggesting that executive function development in late adolescence may still be affected by familial influences in Mexican immigrant families. Intergenerational transmission was the most salient for working memory among the three executive functions assessed. We also found distinct patterns of influence for different executive function abilities in the context of maternal stressors; these stressors were related to adolescents’ executive function, either by altering the intergenerational transmission process or directly influencing adolescent executive function, depending on the type of executive function. Specifically, we found that low foreigner stress was related to stronger associations between mothers’ and adolescents’ working memory. In contrast, more adverse contexts (i.e., high foreigner stress) related to strengthened intergenerational transmission of shifting ability. Instead of moderating the intergenerational transmission of executive function, higher maternal economic stress was directly associated with worse adolescent inhibitory control.

While it has been shown that the development of executive function could continue into young adulthood (e.g., Blakemore & Mills, 2014), little is known about the familial factors that may influence the development of executive function in late adolescence. The current study is one of the first to demonstrate that maternal executive function may still contribute to adolescents’ executive function, particularly working memory, in late adolescence. We found that intergenerational transmission could explain a fair amount of variation (11%) in adolescents’ working memory, even after controlling for factors such as adolescents’ age and mothers’ education. However, our study found no evidence for a main effect in the intergenerational transmission of inhibitory control or shifting in the baseline models. The significant maternal influence on adolescent working memory, but not the other two cognitive functions, may be attributed to the fundamental role of working memory in a variety of higher-order cognitive tasks, such as language comprehension, reasoning, learning, and ability to maintain and pursue goals, which are all common in people’s day-to-day interactions (Engle, 2002; Ericsson & Delaney, 1999). As parents’ executive function can be transmitted to their children via parent–child interactions (Deater-Deckard, 2014), being engaged in a common cognitive task during daily parent–child interactions may mean the intergenerational transmission of maternal working memory is particularly strong. As there is insufficient evidence in the current study to draw definitive conclusions, we encourage future researchers to undertake a deeper examination of the reason why certain types of executive function are more likely to be transmitted from parent to child.

As posited by prior research, the development of adolescent executive function involves transactional processes whereby parent executive function works in conjunction with environmental factors to shape adolescent executive function (Bridgett et al., 2015; Tucker-Drob et al., 2013). The intergenerational transmission of executive function is particularly vulnerable to environmental influences in less advantaged socioeconomic contexts (Tucker-Drob et al., 2013). The current findings suggest that maternal foreigner stress may be one salient environmental factor that modifies the intergenerational transmission of executive function among mother–child dyads from low-income Mexican immigrant families.

The intergenerational transmission of working memory and shifting can both be altered by the foreigner stress perceived by mothers, yet in different ways, underscoring the importance of examining each executive function component on its own rather than considering executive function as a singular construct. The intergenerational link for working memory was augmented when mothers reported low to mean levels of foreigner stress. The intergenerational transmission of shifting, on the other hand, emerged only when mothers perceived high levels of foreigner stress. This finding is consistent with past literature that identified the various types of executive function as separate factors, especially during adolescence (e.g., Friedman et al., 2008; Miyake et al., 2000; Schmeichel & Tang, 2015).

The distinct characteristics of each type of executive function may be key for the different moderating mechanisms we found for working memory transmission versus shifting transmission. Specifically, working memory involves short-term maintenance and manipulation of information (Best & Miller, 2010). The extant literature on working memory and stress has consistently demonstrated that individuals’ working memory would be undermined and even impaired by acute or chronic stress (e.g., Evans & Fuller‐Rowell, 2013; Shields et al., 2016). Individual differences in the performance of working memory are likely to be attenuated in the presence of stress. In fact, a study based on laboratory experiments found that undergraduate students with high working memory adopted more sophisticated algorithms to solve math problems and had higher accuracy than those with low working memory *only* in low-stress contexts but not high-stress contexts (Beilock & DeCaro, 2007). Hence, one possibility is that mothers with low foreigner stress may be better positioned to fully utilize their working memory in their day-to-day activities, such that their working memory may be more likely to be transmitted to their children. Thus, there was a stronger positive link between mothers’ working memory and that of their children when mothers perceived low foreigner stress.

A second type of executive function examined in the study, shifting, is a core component of cognitive flexibility (Diamond, 2013). It is the ability to flexibly switch between behaviors, rules or mental states (Best & Miller, 2010). As shifting helps individuals adapt to their changing situations, it may be that individual differences in shifting ability are more likely to manifest in contexts that require constant switching between rules or mindsets. Although no study has directly examined this speculation, one relevant study found that the shifting advantage of living in an unpredictable childhood environment *only* emerged for undergraduate participants who had a sense of environmental uncertainty (Mittal et al., 2015). We surmise that Mexican immigrant mothers with higher foreigner stress are likely to face or perceive more challenges involving switching between languages and cultural rules, thereby using their shifting skills more often. For example, a mother may need to make sense of a notice written by her landlord in English rather than Spanish, while also shifting attention to parenting her U.S.-born adolescents who possess stronger American cultural values relative to her stronger Mexican cultural values. With more exposure to mothers’ utilization of shifting ability, it is plausible that adolescents would resemble their mothers in terms of shifting ability. As such, it is likely that the intergenerational transmission of shifting was enhanced when mothers experienced higher levels of foreigner stress. More studies are needed to investigate this hypothesis.

In summary, we argue that an executive function skill tends to be transmitted from mothers to adolescents when the environment facilitates the emergence of individual differences in that specific executive function for mothers. While low maternal foreigner stress may allow mothers to fully apply their working memory resources, high maternal foreigner stress may create more opportunities for mothers to utilize their shifting ability. Therefore, the intergenerational transmission of working memory was enhanced when mothers reported lower foreigner stress, whereas the intergenerational transmission of shifting was strengthened when mothers reported higher foreigner stress. The impact of foreigner stress on immigrant families may be more pronounced in the years to come, given the current socio-cultural climate and the rise of anti-immigrant sentiment in the U.S. Future research on the intergenerational transmission of executive function among ethnic minority populations should also consider factors that are culturally relevant, such as foreigner stress.

Interestingly, unlike maternal foreigner stress, maternal economic stress did not demonstrate any moderating effect upon the intergenerational transmission of executive function. We assume that one possible reason for this finding is that the family’s financial responsibilities are typically assumed by the father rather than the mother in traditional Mexican culture (Updegraff et al., 2014). Although the mothers who participated in the current study experienced economic stress, the burden of resolving the financial difficulties behind it may have fallen more upon fathers. Therefore, mothers’ economic stress may have interfered less with the transmission of executive function from mothers to adolescents. It should also be noted that the current study is based on a low-income sample. The moderating effect of economic stress may manifest when there is more variation in participants’ family income.

Rather than moderating the intergenerational transmission of executive function, mothers’ economic stress compromised the development of adolescents’ inhibitory control directly. This is consistent with past research, which found that children from less advantaged socioeconomic backgrounds tend to develop poorer executive function (e.g., Farah et al., 2006). To some extent, maternal economic stress could reflect the family’s financial condition and social welfare. Mothers who perceived more economic stress may have experienced greater difficulty providing their adolescents with the rich, stable, and stimulating environment necessary for optimal cognitive development. Lacking such an environment may be particularly harmful to the development of inhibitory control (Mittal et al., 2015). Inhibitory control is adaptive in the pursuit of long-term goals, as it involves the ability to intentionally suppress dominant responses (Best & Miller, 2010). Families with stronger economic stress may not have consistent instrumental support for the children. Consequently, less inhibition may be an adaptive strategy that allows children to take advantage of fleeting opportunities and rewards in the short term (Mittal et al., 2015).

## Limitations

The current study has several limitations that may compromise the generalizability of its findings. Firstly, it is important to note that the effects are essentially correlational, as the analyses were based on concurrent data. It is unclear whether the concurrent associations would unfold across time in a similar way. Therefore, caution should be taken in making predictions based on the current findings. Future research should examine these associations using longitudinal data. Secondly, while our study has controlled for language proficiency and maternal education as a proxy for intelligence, we did not assess general intelligence (e.g., Barbey et al., 2012). Although there is evidence that the intergenerational transmission of executive function is independent of both parent intelligence (Jester et al., 2009) and adolescent intelligence (Brieant et al., 2017), it is still important to rule out possible confounding effects of intelligence in order to be more confident about the specific effects of executive function. Third, although executive function in our study was measured by well-developed paradigms in experimental settings, future studies are needed to understand the ecological validity of our paradigms in predicting adolescents’ everyday executive function skills (Chaytor et al., 2006). Relatedly, future studies that incorporate multiple informants (e.g., parent and teacher reports) of adolescents’ executive function and/or adopt paradigms that can be applied in a naturalistic setting (e.g., using virtual reality) are needed to understand the ecological validity of our findings.

Finally, although the current study considers the role of economic stress and foreigner stress in the development of adolescent executive functions among Mexican immigrant families, other environmental factors are also worth noting and investigating. Factors related to immigrant status, physical appearance, and language barriers are likely to matter to individuals with a Mexican immigrant background (Kim, Schwartz, et al., 2018b; Zou & Cheryan, 2017). We encourage future research to explore the development of adolescent executive function in contexts that are salient to the target population. For example, research focusing on immigrant adolescents may consider reception and immigration policies, which are increasingly salient cultural contexts for immigrants. In addition, given the heavily gendered family roles assumed by mothers and fathers in traditional Mexican culture (Updegraff et al., 2014), it is likely that the intergenerational transmission of executive function operates differently for mother–child dyads versus father-child dyads. Also, the current sample was limited in terms of participants’ socio-economic background. As the impact of environmental factors on executive function’s intergenerational transmission is likely to be stronger for low-SES families (Tucker-Drob et al., 2013), the associations found in the current study may not be found for families with higher SES. Moreover, our research on environmental factors that are culturally relevant for Mexican Americans, such as foreigner stress, was not tested for other cultural groups, which would have provided a useful contrast to the current findings on intergenerational transmission of executive function. Future studies should include participants with more diverse ethnic and socioeconomic backgrounds for a more comprehensive understanding of the intergenerational transmission of executive function.

## Conclusion

Our findings offer three major contributions to the existing literature. First, our study extends prior research, which has mainly focused on early childhood and data from middle-class White samples (e.g., Deater-Deckard, 2014), by demonstrating the intergenerational transmission of executive function during late adolescence in a sample of low-income Mexican immigrant families. The intergenerational transmission of working memory could be particularly pronounced for the target population of the present study, possibly because working memory has greater vulnerability to the home environment shared by the mother-adolescent dyad. Second, our study suggests that the development of adolescent executive function may be affected differently by maternal foreigner stress and economic stress. In moderating the intergenerational transmission of executive function, stressors that are more specific to ethnic minority groups, such as foreigner stress, may be more powerful than economic stress. Considering the current anti-immigrant sentiment in the U.S., these findings underscore the importance of studying culturally-specific factors in immigrant populations. Finally, our findings indicate that the process of intergenerational transmission, in the context of culturally relevant stress experienced by mothers, operates differently according to the component of executive function being examined, which suggests that executive function should be examined as a multi-dimensional construct rather than a uni-dimensional one.

## Supplementary Information

**Additional file 1: Figure S1.** Violin plot of digit span by participant (adolescent vs. mother). **Note.** The dot in the center indicates the mean, and the vertical line around the dot is the error bar showing one standard deviation above and below the mean. **Figure S2.** Violin plot of Simon effect by participant (adolescent vs. mother). **Note.** The dot in the center indicates the mean, and the vertical line around the dot is the error bar showing one standard deviation above and below the mean. **Figure S3.** Violin plot of switching cost by participant (adolescent vs. mother). **Note.** The dot in the center indicates the mean, and the vertical line around the dot is the error bar showing one standard deviation above and below the mean.

## Data Availability

The datasets used and/or analyzed during the current study are available from the corresponding author on reasonable request.

## References

[CR1] Armenta BE, Lee RM, Pituc ST, Jung K-R, Park IJ, Soto JA, Kim SY, Schwartz SJ (2013). Where are you from? A validation of the Foreigner Objectification Scale and the psychological correlates of foreigner objectification among Asian Americans and Latinos. Cultural Diversity and Ethnic Minority Psychology.

[CR2] Barbey AK, Colom R, Solomon J, Krueger F, Forbes C, Grafman J (2012). An integrative architecture for general intelligence and executive function revealed by lesion mapping. Brain.

[CR3] Bardikoff N, Sabbagh M, Budwig N, Turiel E, Zelazo PD (2017). The differentiation of executive functioning across development: Insights from developmental cognitive neuroscience. New perspectives on human development.

[CR4] Beilock SL, DeCaro MS (2007). From poor performance to success under stress: Working memory, strategy selection, and mathematical problem solving under pressure. Journal of Experimental Psychology: Learning, Memory, and Cognition.

[CR5] Bernier A, Carlson SM, Whipple N (2010). From external regulation to self-regulation: Early parenting precursors of young children’s executive functioning. Child Development.

[CR6] Best JR, Miller PH (2010). A developmental perspective on executive function. Child Development.

[CR7] Bialystok E (2011). Reshaping the mind: The benefits of bilingualism. Canadian Journal of Experimental Psychology.

[CR8] Bialystok E, Craik FIM, Klein R, Viswanathan M (2004). Bilingualism, aging, and cognitive control: Evidence from the Simon task. Psychology and Aging.

[CR9] Blakemore S-J, Mills KL (2014). Is adolescence a sensitive period for sociocultural processing?. Annual Review of Psychology.

[CR10] Bridgett DJ, Burt NM, Edwards ES, Deater-Deckard K (2015). Intergenerational transmission of self-regulation: A multidisciplinary review and integrative conceptual framework. Psychological Bulletin.

[CR11] Brieant A, Holmes CJ, Deater-Deckard K, King-Casas B, Kim-Spoon J (2017). Household chaos as a context for intergenerational transmission of executive functioning. Journal of Adolescence.

[CR12] Chaytor N, Schmitter-Edgecombe M, Burr R (2006). Improving the ecological validity of executive functioning assessment. Archives of Clinical Neuropsychology.

[CR13] Choudhury S, Blakemore S-J, Charman T (2006). Social cognitive development during adolescence. Social Cognitive and Affective Neuroscience.

[CR14] Conger RD, Donnellan MB (2007). An interactionist perspective on the socioeconomic context of human development. Annual Review of Psychology.

[CR15] Conger RD, Wallace LE, Sun Y, Simons RL, McLoyd VC, Brody GH (2002). Economic pressure in African American families: A replication and extension of the family stress model. Developmental Psychology.

[CR16] Deater-Deckard K (2014). Family matters: Intergenerational and interpersonal processes of executive function and attentive behavior. Current Directions in Psychological Science.

[CR17] Deater-Deckard K, Chen N, Wang Z, Bell MA (2012). Socioeconomic risk moderates the link between household chaos and maternal executive function. Journal of Family Psychology.

[CR18] Della Sala S, Foley JA, Beschin N, Allerhand M, Logie RH (2010). Assessing dual-task performance using a paper-and-pencil test: Normative data. Archives of Clinical Neuropsychology.

[CR19] Diamond A (2013). Executive functions. Annual Review of Psychology.

[CR20] Dunn AL, Fox Tree JE (2009). A quick, gradient Bilingual Dominance Scale. Bilingualism: Language and Cognition.

[CR21] Ellis BJ, Bianchi J, Griskevicius V, Frankenhuis WE (2017). Beyond risk and protective factors: An adaptation-based approach to resilience. Perspectives on Psychological Science.

[CR22] Engle RW (2002). Working memory capacity as executive attention. Current Directions in Psychological Science.

[CR23] Ericsson KA, Delaney PF, Miyake A, Shah P (1999). Long-term working memory as an alternative to capacity models of working memory in everyday skilled performance. Models of working memory: Mechanisms of active maintenance and executive control.

[CR24] Evans GW, Fuller-Rowell TE (2013). Childhood poverty, chronic stress, and young adult working memory: The protective role of self-regulatory capacity. Developmental Science.

[CR25] Farah MJ, Shera DM, Savage JH, Betancourt L, Giannetta JM, Brodsky NL, Malmud EK, Hurt H (2006). Childhood poverty: Specific associations with neurocognitive development. Brain Research.

[CR26] Friedman NP, Miyake A, Young SE, DeFries JC, Corley RP, Hewitt JK (2008). Individual differences in executive functions are almost entirely genetic in origin. Journal of Experimental Psychology: General.

[CR27] Heitzeg MM, Cope LM, Martz ME, Hardee JE (2015). Neuroimaging risk markers for substance abuse: Recent findings on inhibitory control and reward system functioning. Current Addiction Reports.

[CR28] Hilchey MD, Klein RM (2011). Are there bilingual advantages on nonlinguistic interference tasks? Implications for the plasticity of executive control processes. Psychonomic Bulletin & Review.

[CR29] Jester JM, Nigg JT, Puttler LI, Long JC, Fitzgerald HE, Zucker RA (2009). Intergenerational transmission of neuropsychological executive functioning. Brain and Cognition.

[CR30] Kim MH, Shimomaeda L, Giuliano RJ, Skowron EA (2017). Intergenerational associations in executive function between mothers and children in the context of risk. Journal of Experimental Child Psychology.

[CR31] Kim SY, Hou Y, Song J, Schwartz SJ, Chen S, Zhang M, Perreira KM, Parra-Medina D (2018). Profiles of language brokering experiences and contextual stressors: Implications for adolescent outcomes in Mexican immigrant families. Journal of Youth and Adolescence.

[CR32] Kim SY, Schwartz SJ, Perreira KM, Juang LP (2018). Culture’s influence on stressors, parental socialization, and developmental processes in the mental health of children of immigrants. Annual Review of Clinical Psychology.

[CR33] Kim SY, Zhang M, Chen S, Song J, Lopez BG, Rodriguez EM, Calzada EJ, Hou Y, Yan J, Shen Y (2020). Bilingual language broker profiles and academic competence in Mexican-origin adolescents. Developmental Psychology.

[CR34] Lawson GM, Hook CJ, Farah MJ (2018). A meta-analysis of the relationship between socioeconomic status and executive function performance among children. Developmental Science.

[CR35] Lee K, Bull R, Ho RMH (2013). Developmental changes in executive functioning. Child Development.

[CR36] Lê-Scherban F, Diez Roux AV, Li Y, Morgenstern H (2014). Does academic achievement during childhood and adolescence benefit later health?. Annals of Epidemiology.

[CR37] Lewis C, Carpendale JIM (2009). Social interaction and the development of executive function. New Directions for Child and Adolescent Development.

[CR38] Li J, Zhao Y, Zhou S, Pu Y, He H, Zhao M (2020). Set-shifting ability is specifically linked to high-school science and math achievement in Chinese adolescents. PsyCh Journal.

[CR39] Little RJ (1998). A test of missing completely at random for multivariate data with missing values. Journal of the American Statistical Association.

[CR40] Lopez, M. H., & Velasco, G. (2011). *Childhood poverty among Hispanics sets record, leads nation*. Pew Research Center. Retrived from, https://www.pewresearch.org/hispanic/2011/09/28/childhood-poverty-among-hispanics-sets-record-leads-nation/#:~:text=According%20to%20the%202010%20U.S.,of%20the%20total%20U.S.%20population.&text=Of%20the%206.1%20million%20Latino,new%20Pew%20Hispanic%20Center%20analysis.

[CR41] Luethi M, Meier B, Sandi C (2009). Stress effects on working memory, explicit memory, and implicit memory for neutral and emotional stimuli in healthy men. Frontiers in Behavioral Neuroscience.

[CR42] Macnamara BN, Conway AR (2016). Working memory capacity as a predictor of simultaneous language interpreting performance. Journal of Applied Research in Memory and Cognition.

[CR43] Malagoli C, Usai MC (2018). The effects of gender and age on inhibition and working memory organization in 14- to 19-year-old adolescents and young adults. Cognitive Development.

[CR44] Mittal C, Griskevicius V, Simpson JA, Sung S, Young ES (2015). Cognitive adaptations to stressful environments: When childhood adversity enhances adult executive function. Journal of Personality and Social Psychology.

[CR45] Miyake A, Emerson MJ, Padilla F, Ahn J-C (2004). Inner speech as a retrieval aid for task goals: The effects of cue type and articulatory suppression in the random task cuing paradigm. Acta Psychologica.

[CR46] Miyake A, Friedman NP, Emerson MJ, Witzki AH, Howerter A (2000). The unity and diversity of executive functions and their contributions to complex 'frontal lobe' tasks: A latent variable analysis. Cognitive Psychology.

[CR47] Muthén, L. K., & Muthén, B. O. (1998-2012). *Mplus user’s guide* (7th ed.). Muthén & Muthén.

[CR48] Ozier EM, Taylor VJ, Murphy MC (2019). The cognitive effects of experiencing and observing subtle racial discrimination. Journal of Social Issues.

[CR49] Raver CC, Blair C, Willoughby M (2013). Poverty as a predictor of 4-year-olds' executive function: New perspectives on models of differential susceptibility. Developmental Psychology.

[CR50] Rigoli D, Piek JP, Kane R, Oosterlaan J (2012). Motor coordination, working memory, and academic achievement in a normative adolescent sample: Testing a mediation model. Archives of Clinical Neuropsychology.

[CR51] Schmeichel BJ, Tang D (2015). Individual differences in executive functioning and their relationship to emotional processes and responses. Current Directions in Psychological Science.

[CR52] Schneider W, Eschman A, Zuccolotto A (2002). E-Prime 2.0.

[CR53] Shields GS, Sazma MA, Yonelinas AP (2016). The effects of acute stress on core executive functions: A meta-analysis and comparison with cortisol. Neuroscience and Biobehavioral Reviews.

[CR54] Simon JR, Rudell AP (1967). Auditory S-R compatibility: The effect of an irrelevant cue on information processing. Journal of Applied Psychology.

[CR55] Sosic-Vasic Z, Kröner J, Schneider S, Vasic N, Spitzer M, Streb J (2017). The association between parenting behavior and executive functioning in children and young adolescents. Frontiers in Psychology.

[CR56] Theodoraki TE, McGeown SP, Rhodes SM, MacPherson SE (2019). Developmental changes in executive functions during adolescence: A study of inhibition, shifting, and working memory. British Journal of Developmental Psychology.

[CR57] Tienda M, Haskins R (2011). Immigrant children: Introducing the issue. The Future of Children.

[CR58] Tottenham N, Galván A (2016). Stress and the adolescent brain: Amygdala-prefrontal cortex circuitry and ventral striatum as developmental targets. Neuroscience and Biobehavioral Reviews.

[CR59] Tucker-Drob EM, Briley DA, Harden KP (2013). Genetic and environmental influences on cognition across development and context. Current Directions in Psychological Science.

[CR60] Tymula A, Belmaker LAR, Roy AK, Ruderman L, Manson K, Glimcher PW, Levy I (2012). Adolescents' risk-taking behavior is driven by tolerance to ambiguity. Proceedings of the National Academy of Sciences of the United States of America.

[CR61] Updegraff KA, McHale SM, Zeiders KH, Umaña-Taylor AJ, Perez-Brena NJ, Wheeler LA, Rodríguez De Jesús SA (2014). Mexican-American adolescents’ gender role attitude development: The role of adolescents’ gender and nativity and parents’ gender role attitudes. Journal of Youth and Adolescence.

[CR62] Wechsler DA (1997). Wechsler memory scale (WMS-III).

[CR63] Weintraub S, Dikmen SS, Heaton RK, Tulsky DS, Zelazo PD, Bauer PJ, Carlozzi NE, Slotkin J, Blitz D, Wallner-Allen K, Fox NA, Beaumont JL, Mungas D, Nowinski CJ, Richler J, Deocampo JA, Anderson JE, Manly JJ, Borosh B, Havlik R, Conway K, Edwards E, Freund L, King JW, Moy C, Witt E, Gershon RC (2013). Cognition assessment using the NIH Toolbox. Neurology.

[CR64] White RMB, Roosa MW, Weaver SR, Nair RL (2009). Cultural and contextual influences on parenting in Mexican American families. Journal of Marriage and Family.

[CR65] Williams AI, Uchikoshi Y, Bunge SA, Zhou Q (2019). Relations of English and heritage language proficiency to response inhibition and attention shifting in dual language learners in head start. Early Education and Development.

[CR66] Zou LX, Cheryan S (2017). Two axes of subordination: A new model of racial position. Journal of Personality and Social Psychology.

