# Supplementary material for: Culturally relevant stressors as moderators of intergenerational transmission of mother-adolescent executive function in Mexican immigrant families
Source: Cogn Res Princ Implic. 2021 Nov 3;6:70. doi: 10.1186/s41235-021-00333-x (PMC8566615; doi:10.1186/s41235-021-00333-x)
Supplement: Supplementary file 1 — Additional file 1: Figure S1. Violin plot of digit span by participant (adolescent vs. mother). Note. The dot in the center indicates the mean, and the vertical line around the dot is the error bar showing one standard deviation above and below the mean. Figure S2. Violin plot of Simon effect by participant (adolescent vs. mother). Note. The dot in the center indicates the mean, and the vertical line around the dot is the error bar showing one standard deviation above and below the mean. Figure S3. Violin plot of switching cost by participant (adolescent vs. mother). Note. The dot in the center indicates the mean, and the vertical line around the dot is the error bar showing one standard deviation above and below the mean. [file 41235_2021_333_MOESM1_ESM.docx]

**Appendix**

**Figure S1**

*Violin plot of digit span by participant (adolescent vs. mother)*


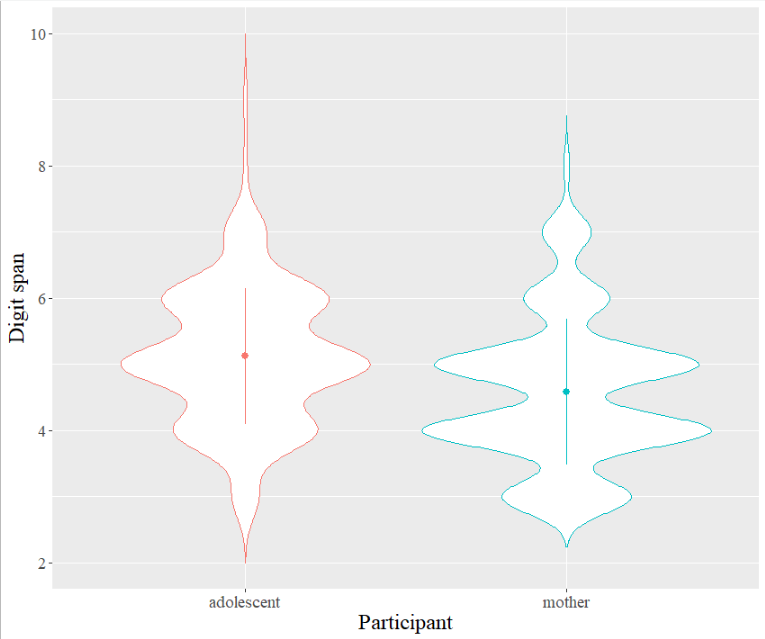


*Note.* The dot in the center indicates the mean, and the vertical line around the dot is the error bar showing one standard deviation above and below the mean.

**Figure S2**

*Violin plot of Simon effect by participant (adolescent vs. mother)*


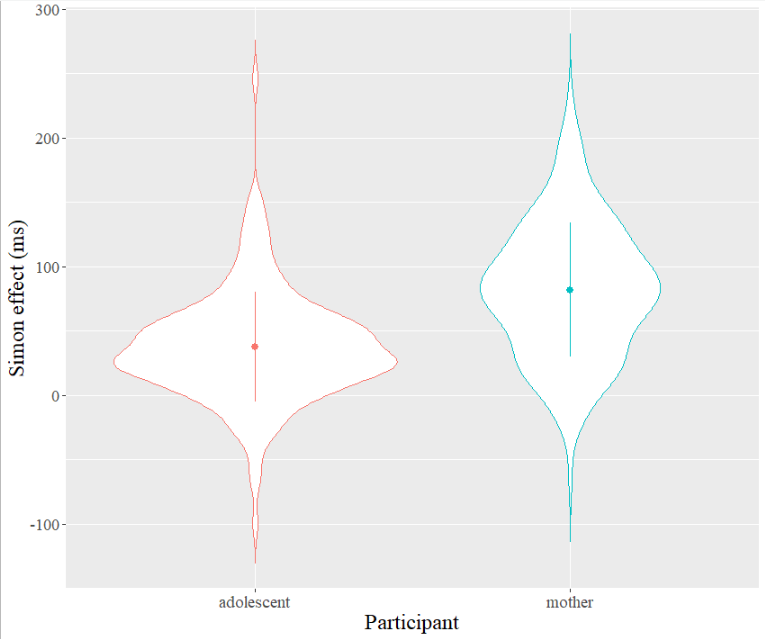


*Note.* The dot in the center indicates the mean, and the vertical line around the dot is the error bar showing one standard deviation above and below the mean.

**Figure S3**

*Violin plot of switching cost by participant (adolescent vs. mother)*


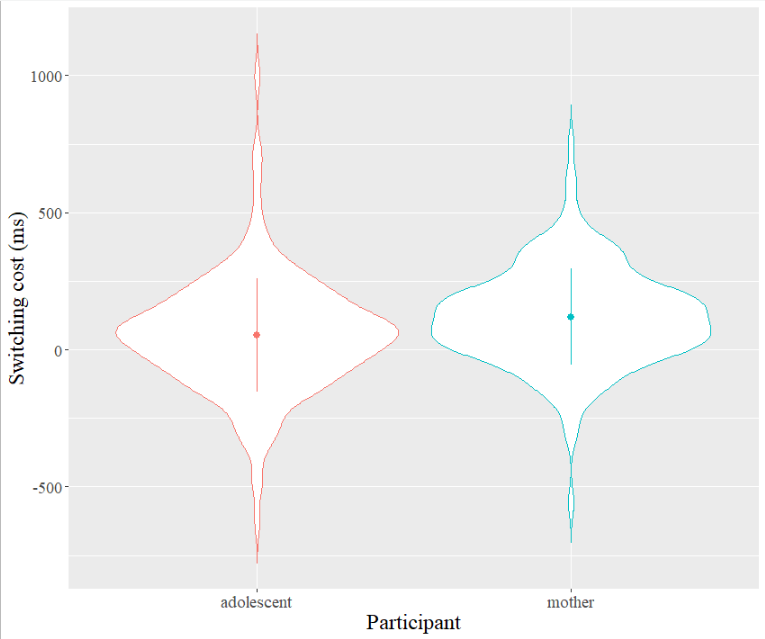


*Note.* The dot in the center indicates the mean, and the vertical line around the dot is the error bar showing one standard deviation above and below the mean.
